# Oral Storytelling as Evidence of Pedagogy in Forager Societies

**DOI:** 10.3389/fpsyg.2017.00471

**Published:** 2017-03-29

**Authors:** Michelle Scalise Sugiyama

**Affiliations:** Department of Anthropology, University of OregonEugene, OR, USA

**Keywords:** natural pedagogy, oral narrative, ostensive communication, social learning, cultural transmission, hunter-gatherers, paralinguistic communication

## Abstract

Teaching is reportedly rare in hunter-gatherer societies, raising the question of whether it is a species-typical trait in humans. A problem with past studies is that they tend to conceptualize teaching in terms of Western pedagogical practices. In contrast, this study proceeds from the premise that teaching requires the ostensive manifestation of generalizable knowledge: the teacher must signal intent to share information, indicate the intended recipient, and transmit knowledge that is applicable beyond the present context. Certain features of human communication appear to be ostensive in function (e.g., eye contact, pointing, contingency, prosodic variation), and collectively serve as “natural pedagogy.” Tellingly, oral storytelling in forager societies typically employs these and other ostensive behaviors, and is widely reported to be an important source of generalizable ecological and social knowledge. Despite this, oral storytelling has been conspicuously overlooked in studies of teaching in preliterate societies. Accordingly, this study presents evidence that oral storytelling involves the use of ostension and the transmission of generic knowledge, thereby meeting the criteria of pedagogy.

## The role of knowledge in the human ecological niche

Recent years have seen renewed interest in the question of whether teaching is a species-typical trait in humans (Hewlett and Roulette, 2016). This question arises from the nature of our ecological niche. Humans are designed to make their living by foraging, which requires extensive, detailed knowledge of plant properties and growth habits, animal behavior, weather patterns, topography, wayfinding, and tool use and manufacture, among other things (Boyd et al., 2011). Indeed, indigenous knowledge of local ecosystems and their seasonal and annual fluctuations is so nuanced and reliable that it has been recognized as an important source of data by environmental scientists and public policy makers. For example, the traditional ecological knowledge (TEK) of Cree and Inuit peoples was instrumental in assessing the actual and potential environmental impacts of development in the Hudson Bay bioregion (McDonald et al., 1997). Thus, while other species have evolved anatomical features such as speed, strength, camouflage, and keen sensory systems that aid in their survival, humans depend for their living on the ability to extract and apply information (Tooby and DeVore, 1987). Humans are also highly dependent on the ability to generate new knowledge through exploration, experimentation, and inference. Known as *improvisational intelligence* (Barrett et al., 2007), this ability enables humans to compensate for their anatomical limitations by inventing tools and tactics that allow them to access resources that would otherwise be unavailable to them (Kaplan et al., 2000).

One of the challenges presented by our information-dependent niche is the timely acquisition of knowledge. Although the extended human juvenile period is believed to have evolved to support learning as well as growth (Lancaster and Lancaster, 1983; Kaplan et al., 2000), the depth and breadth of knowledge demanded by the foraging niche cannot be acquired solely through first-hand experience by the time it is needed in early adulthood (Boyd et al., 2011). One solution that appears to have emerged in response to this problem is information sharing, which greatly reduces the costs and risks of knowledge acquisition. Information sharing eliminates dependence on the occurrence of suitable learning opportunities, exponentially increasing the amount of knowledge an individual can acquire and the rate at which it can be acquired (Scalise Sugiyama, 2016), and ultimately enabling social groups to accumulate vast stores of collective knowledge (Tomasello, 1999). Quantitative research among forager populations indicates that the knowledge and skills upon which survival depends are acquired primarily from conspecifics (Hewlett and Cavalli-Sforza, 1986; Ohmagari and Berkes, 1997). However, the degree to which instruction occurs in forager populations, as well as the mechanisms of instruction, are largely unknown.

## Western teaching vs. natural pedagogy

The intense information demands of the foraging niche raise questions about the behavioral mechanics of information transmission. Forager, forager-horticulturalist, and forager-pastoralist (henceforth “forager”) societies are an important source of data on this question, because their living conditions approximate those under which our species evolved. Although modern foragers differ from ancestral foragers in (among other things) their access to metal, use of dogs, and occupation of marginal habitats, the congruencies between them are significant. In both cases, subsistence depends on the harvesting of wild foods; social organization is based largely on small, egalitarian, nomadic or semi-nomadic bands with a pronounced sexual division of labor; and available technology does not include labor- and life-saving developments such as motorized transport, telecommunication, and Western medicine (Lee and DeVore, 1968; Marlowe, 2005). Due to these pronounced socio-ecological similarities, the study of modern foraging peoples is critical to reconstructing the selection pressures that shaped the design of our species (Tooby and Cosmides, 1990).

As noted above, one of these selection pressures was knowledge acquisition: individuals incapable of acquiring sufficient local ecological knowledge by early adulthood would have been at elevated risk of starvation, exposure, predator attack, and a host of other hazards. Despite the obvious fitness benefits of rapid and efficient knowledge acquisition, several researchers allege that teaching is rare in forager societies (e.g., Gould, 1969; Lee, 1969; Blurton Jones and Konner, 1976; MacDonald, 2007). For example, Nelson reports that the Kutchin “train their own providers by the active participation technique. Young men are not given verbal instruction; they watch, try for themselves, then are corrected for their mistakes” (Nelson, 1973,p. 9). However, many of these claims conceptualize teaching in terms of Western pedagogical practices (Lancy, 2010; Rogoff, 2011), and are belied by studies showing that teaching and learning are not limited to formal classroom instruction (e.g., Greenfield and Lave, 1982), and others showing that foragers acquire practical skills almost exclusively from conspecifics (Hewlett and Cavalli-Sforza, 1986; Ohmagari and Berkes, 1997). The latter suggest that social learning plays an important role in forager knowledge acquisition; however, they are few in number and focus on skills rather than the zoological, botanical, and other knowledge sets that scaffold them. On this point, oral tradition is widely characterized as an important source of both ecological and social knowledge in forager societies (e.g., Opler, 1938; Ramsey, 1977; McClellan, 1987; Burch, 1991; Biesele, 1993; de Laguna, 1995; Basso, 1996). Considerable knowledge of animal behavior, for example, is acquired by listening to accounts of hunting excursions that are shared among hunters in camp (Leacock, 1954, p. 14; Laughlin, 1968, p. 308; Nelson, 1969, p. 374; Blurton Jones and Konner, 1976; Biesele, 1978, p. 940; Ridington, 1988, p. 47; Kaplan and Hill, 1992, p. 196). To date, however, this material has not been examined as evidence of teaching in forager populations.

The question of whether humans are a teaching species hinges on the definition of teaching, which varies across disciplines. Cognitive psychologists define teaching as “a behavior in which one animal intends that another learn some skill or acquire some bit of information or knowledge that it did not have previously” (Kruger and Tomasello, 1996, p. 374). Behavioral biologists argue that teaching occurs when (a) an expert modifies its behavior (b) only in the presence of a novice, and (c) the expert incurs a cost (or derives no immediate benefit), while (d) the novice benefits by acquiring skills or knowledge that it would not acquire otherwise or would acquire less efficiently (Caro and Hauser, 1992, p. 153). Taking a design-based approach, Csibra and Gergely (2006, 2009) argue that certain features of human communication appear to be specialized for intraspecific transfer and acquisition of generic knowledge; collectively, these mechanisms constitute a “natural pedagogy” characterized by the ostensive manifestation of knowledge by the expert and the recognition of this manifestation by the novice. Borrowing Sperber and Wilson's (1986) concept of *ostensive communication*, Csibra and Gergely argue that pedagogy is not just a matter of dispensing knowledge: in order for teaching to occur, the expert must indicate that she is manifesting her knowledge and must indicate the intended recipient of that knowledge. Ostension thus includes behaviors such as eye contact, pointing, contingency, and modification of prosody. They further argue that, in contrast to non-human animal communication, natural pedagogy is designed to transmit generalizable knowledge: information that is applicable outside of the present location, occasion, and/or context.

Oral storytelling appears to meet all of the above definitions in that it involves the modification of behavior by an expert to enable or facilitate information acquisition by one or more novices. The “expert” in this case is the storyteller, whose expertise comprises a corpus of tales and the information encoded therein. The “modification of behavior to facilitate information acquisition” is the act of telling a story. The “novices” are those audience members who are unfamiliar with the story or whose memory of it has faded. With respect to storytelling, then, claims of pedagogy rest on demonstrating that storytellers modify their behavior in ways that enable or facilitate learning in others, and that storytelling communicates generalizable knowledge. By specifying the design features of pedagogy, Csibra and Gergely's model provides criteria that can be used to test these claims. Accordingly, the remainder of this essay reviews evidence that oral storytelling involves the use of ostensive communication and the transmission of generic knowledge. In so doing, it provides a new line of evidence for the existence of natural pedagogy.

## Use of ostension in oral storytelling

In order to teach, an individual must signal pedagogical intent: the individual's behavior must be marked as a pedagogical act so that the intended recipient(s) will pay attention. This can be accomplished through visual, auditory, and/or gestural means (Csibra and Gergely, 2006, 2009). All three of these modes are used in oral storytelling, but have escaped the attention of researchers involved in the natural pedagogy debate. This may be due to disciplinary insularity between biology-based social scientists and ethno-linguists, compounded by their different lexica: what cognitive psychologists refer to as *ostensive communication* is referred to as the *paralinguistic features of communication* by ethno-linguists.

The most obvious visual cue of pedagogical intent is direct gaze toward the addressee. Significantly, storytelling is characterized by mutual gazing, with the storyteller facing the audience and the audience facing the storyteller. Much more prominent in oral narration, however, is the use of auditory cues. These cues bear a marked resemblance to infant-directed speech, or the modification of prosody by adults when speaking to pre-verbal infants. Known as “motherese,” these modifications include higher pitch, exaggerated pitch excursions, broader pitch and amplitude variation, slower speed, longer pauses, and increased repetition (Fernald, 1985). Among other things, these modifications are believed to attract attention and direct it to new information. However, as Csibra and Gergely (2006) emphasize, these modifications also signal to infants that an utterance is directed at them.

Storytellers similarly modify their prosody in ways that differ from everyday speech. Mohave myths, for example, “are told in an almost ritualized style” (Kroeber, 1948, p. 1), and Wiessner reports that, among the Ju/hoansi, the “language of stories tended to be rhythmic” (2014, p. 14029). Compared to ordinary speech, storytelling is characterized by greater and more frequent variation in volume, tempo, timbre, pitch, and stress. The voice may be lowered to a whisper or raised to a shout. For example, Zuñi oral narrative alternates between loud and soft sentences (Tedlock, 1971), and among the Dena, “the story-teller speaks slowly, in a sort of mysterious undertone, which contributes… to cast a sort of awe on the audience” (Jetté, 1908–1909, p. 298). The pace of narration may increase, slow down, or even pause momentarily. Indeed, pauses are one of the most characteristic features of oral narrative, and vary in length: Tedlock's (1971, p. 127) analysis of tape-recorded Zuñi oral narratives found that pauses ranged from four-tenths of a second to 3 s. Pauses are typically used to break long sentences into smaller units, but are also used for emphasis (Tedlock, 1977).

Prosody is also modified by the inclusion of singing, chanting, or other musical accompaniment. For example, Iñupiaq storytellers “frequently interject songs into their presentations, sometimes with drum accompaniment” (Burch, 2005, p. 50). Among the Miwok, “not infrequently the myth was chanted,” and “each myth, whether chanted or told in ordinary prose, was accompanied by the songs of the various characters. For example, with the story of Prairie Falcon's Marriage belong three songs, one sung by Prairie Falcon, one by his wife, and one by his father” (Gifford, 1917, p. 284). Similarly, Mohave stories were told in units or episodes, each of which was punctuated by one or more songs, for a total of 100–400 songs per story (Kroeber, 1948, p. 2). According to Sapir, there is “one type of myth-song that is evidently very common in America. This is the short song found inserted here and there in the body of a myth, generally intended to express some emotion or striking thought of a character” (1910, p. 455). Singing is also used to reinforce theme (Kroeber, 1948, p. 2), and may increase salience by adding meter and intensifying mood, as in oral epic poetry (Lord, 1960, p. 4). Yuman and Shoshone myths are a case in point: these stories feature “a heavy embroidery of songs which carry an emotional stimulus of their own, and at the same time endow the plot with… a feeling” (Kroeber, 1948, p. 1). The use of song in conjunction with storytelling has also been documented among southwestern Oregon Athabaskans (Seaburg, 2007, p. 58), the Karok (Kroeber and Gifford, 1980, p. 108), Southern Paiute (Sapir, 1910), Tanaina (Tenenbaum and McGary, 1984), Alaskan Inuit (Ostermann, 1952, p. 159,175, 185, 187), Menomini (Skinner and Satterlee, 1915, p. 236), Bororo (Wilbert and Simoneau, 1983, p. 68), Ainu (Howell, 1951, pp. 361–362); San (Lewis-Williams, 2000, pp. 31–32), Mbuti (Turnbull, 1959, p. 60), and Australian aborigines (Berndt and Berndt, 1994).

Timbre, too, is frequently modified, often to effect the voices of different characters or animals (e.g., McClellan, 1987, p. 248). Vocal mimicry of animal and other ambient sounds is common in oral storytelling, as is the use of onomatopoeia (Opler, 1938, p. xviii; Tedlock, 1971; Blurton Jones and Konner, 1976; Ramsey, 1977; Wilbert and Simoneau, 1990; Napaljarri and Cataldi, 1994, p. 21). For example, stylistic effects used by the Cree include “stage whispers for eerie situations, onomatopoeia and outright imitations of animal sounds” (Ellis, 1995, p. xiv). Prosodic modifications may also include stress shift and vowel lengthening, which “are more frequent in narrative” than in everyday speech (Tedlock, 1971, p. 121). The voice may also be altered to express the physical or emotional state of a character. For example, among the Zuñi, “When a character is trying to pull some tough blades loose from a yucca plant, the narrator may render ‘He pulled’ with the strain of someone who is trying to speak while holding his breath during great exertion,” or may render intense emotion with “a break in his voice, as if he felt like weeping” (Tedlock, 1971, p. 127). Among the Yukon, “Good storytellers usually change their voices to suit the characters or act out parts of the story, and this makes it more interesting and exciting to hear the stories than to read them” (McClellan, 1987, p. 248). Similarly, Maidu “tales were commonly told by assuming different voices for different characters, thus further enhancing the theatrical effect of the performance” (Shipley, 1991, p. 178).

Pedagogical intent can also be signaled through body language, as seen in the modifications made by adults when demonstrating actions to infants, known as “motionese” (Brand et al., 2002). Compared to adult-directed demonstration, infant-directed demonstration exhibits greater interactiveness, range of motion, repetitiveness, simplicity, and enthusiasm, the latter of which is conveyed through facial expressions and amplified body movements. Although storytelling does not often involve how-to demonstrations, it does prominently feature the use of body movement and facial expression, which (as in motionese) tend to be exaggerated. Narrators frequently mime the actions and expressions of story characters (Opler, 1938, p. xviii; Napaljarri and Cataldi, 1994, p. 21), and use hand and body gestures for punctuation, emphasis, deixis, demonstration, and to indicate travel or time lapse (Turnbull, 1959, p. 60; see photos in Scheub, 1977). Iñupiaq storytellers, for example, “make extensive use of hand, arm, and body gestures; they laugh, cry, smile, and frown as appropriate” (Burch, 2005, p. 50). In a performance of a Xhosa tale in which a woman defends herself against a monster by throwing her possessions at it, “repeated gestures accompany the words: the demands of the monster, the woman's fears and deepening terror, the throwing of the bits of baggage, the eating movements, the flight—all are mimed” (Scheub, 1977, p. 349). Most transcriptions of oral tales omit this information, but in some cases the narrator's gestures are indicated in brackets—for example, “That other person lay there in the boat, like this [with his arm over his eyes]” (Seaburg, 2007, p. 62). As with speech, the tempo and amplitude of gesticulation may be varied over the course of a performance: “Gestures may become muted… then become more frequent and obvious as the narrative becomes more dramatic. Supplementary movements become more pronounced during crises and climaxes, as words are delivered at greater speed, as the tempo quickens” (Scheub, 1977, p. 369). Research shows that infants prefer motionese to adult-directed demonstrations (Brand and Shallcross, 2008). This may explain why good storytellers intuitively use these techniques to grab and hold audience attention, and why “tales that embodied knowledge or moral precepts were more easily remembered by impressionable young people than direct admonitions by their parents, or long expositions” (de Laguna, 1995, p. 75).

Like infant-directed speech and action, oral storytelling is also characterized by the use of redundancy: patterned repetition of words, phrases, and episodes is one of the most pervasive features of performed narrative (Scheub, 1977; Tedlock, 1977). One manifestation of this phenomenon is the use of formulaic expressions (Lord, 1960) that are rarely if ever used in everyday speech (Tedlock, 1971). On this point, set phrases are frequently used to indicate that a story is about to begin or that it has come to an end (e.g., Deloria, 1932; Clark, 1966, p. 102). Trickster tales, for example, commonly begin with a reference to his perpetual rambling, such as “Coyote was going there” (Ramsey, 1977) or “Then Crow goes again” (McClellan, 1987, p. 254). Dena stories typically concluded with a set phrase indicating that the narrator had shortened the winter by making the time pass quickly—for example, “A part of the winter is become short” (Jetté, 1908–1909, p. 470). These phrases have the effect of signaling to the audience that a pedagogical manifestation is about to begin, or that a pedagogical event has concluded—especially when, as in Zuñi storytelling, they are used exclusively as narrative frames (Tedlock, 1971, p. 123).

Repetition is also widely used for emphasis (Biesele, 1993, p. 25). This may reflect a conscious or unconscious effort on the part of the narrator to call attention to important pedagogical moments in the narrative. Various devices are used, including lexical reduplication (e.g., “her belly got big, big”) and parallel structure (Jacobs, 1972). According to Tedlock, oral storytellers “frequently keep a story in motion by combining strings of clauses into long sentences, and by joining these sentences with parallelism” (1971, p. 119). This technique is seen in a Zuñi story: “They laid the deer down side by side. They laid them down side by side and they made the boy sit down beside them. After they had made him sit down they gave the deer smoke. After they had given them smoke they sprinkled prayer meal on them” (Bunzel, 1933, p. 109). Similarly, many Native American stories “are patterned in terms of lines and groups of lines” (Hymes, 1990, p. 343). For example, five is an important pattern number in Wishram (Hymes, 1990, p. 343) and southwestern Oregon Athabaskan tales (Seaburg, 2007, p. 52). Such devices are typically explained as mnemonics that aid the narrator, but they may serve this function for the audience as well, and buy them time to absorb story content. For example, in Mohave storytelling, “What is going to happen is discussed first, and then it is told over again as a happening. There are arguments between personages on whether to do this or that; whether to understand an event in one way or in another; or as to what is going to happen later” (Kroeber, 1948, p. 3).

Yet another cue of ostension is contingency—behavior that is contingent on the actions of another agent, such as turn-taking in conversation. Contingent responsivity is another common feature of traditional oral storytelling. For example, according to a Wasco informant, a storyteller might “suddenly break off with an interrogatory grunt, ‘Unnhhh?’—i.e., *Are you following?* And the audience, so long as it wanted more, would respond emphatically, ‘Nunnnhhh’—*Yes, keep going!*” (Ramsey, 1977, p. xxv; emphasis in original). Among the Dena, a “narrator would pause politely after the first sentence, until encouraged by the cries of ‘*Anni!*’ from the audience” (de Laguna, 1995, p. 73). The audience might signal that it was paying attention with “peals of laughter, exclamations of commiseration or disgust, reflections on the characters and actions described, [or] conjectures as to what is going to follow” (Jetté, 1908–1909pp. 298–299). Similarly, among the Crow, “the audience was required to answer ‘ε’ (yes) after every sentence or two; when no one replied, it was a sign that all had fallen asleep and the storyteller broke off his/her narrative” (Lowie, 1918, p. 13). Among the Lummi, audience members “participate by interjecting enthusiastic comments to make the exploits of the ancestors appear more vivid” (Stern, 1934, p. 17). The call-and-response technique combines contingency with repetition. Wiessner, for example, observed Ju/hoan audience members “repeating the last words of phrases or adding an affirmative ‘Eh he”’ (2014, p. 14029). Similarly, based on his observation of 10,000 oral narrative performances, Scheub reports that audience members “take up the repeated patterning, with their bodies, voices, and emotions” (1977, p. 345). A Gitksan informant describes a variation on this strategy: “‘when Granny’s telling us a story… I think [s]he deliberately stops and then—“Oh, I forgot the name of that place.” And then somebody volunteers, “Granny, hey…” so. And I think that's just her way of seeing if we remember.… I think that's a little test”' (Johnson, 2010, p. 43). This last observation suggests that storytellers transmit not only generic knowledge, but information about how to transmit generic knowledge.

The reliance of oral storytelling on contingency—and thus its fundamentally pedagogical nature—is revealed in one of the problems faced by linguists when collecting traditional tales: indigenous informants who are bilingual typically want to tell the story in the investigator's own language “so that the investigator will *understand* and *respond*” (Tedlock, 1977, p. 509, emphasis in original). Oral narrative is inherently responsive and participatory; stories are often told in response to a question (Biesele, 1993, p. 58) or event (Tedlock, 1977, p. 515). For example, among the Quiché Maya, “stories occur to people… when conversation or chance events bring them to mind.… in the midst of a conversation about crocodiles and iguanas, someone says, ‘Well, there's a story about that,’ and proceeds to tell it on the spot” (Tedlock, 1977, p. 515). Further evidence of the pedagogical intent of storytelling is the tendency of narrators to “become impatient with nonparticipation” (Tedlock, 1977, p. 515), as seen in a query posed by one of Tedlock's informants: “When I tell a story, do you see it, or do you just write it down?” (1977, p. 515).

Finally, another way to signal pedagogic intent is to establish a teaching context. On this point, many North American forager groups have rules for when stories may and may not be told. These rules are strikingly similar across cultures: storytelling is typically proscribed during the daytime and/or summer months; consequently, in many groups, stories were customarily told on winter nights (e.g., Lowie, 1918, p. 13 [Crow]; Parsons, 1929 [Kiowa]; Deloria, 1932 [Dakota]; Steward, 1936, p. 357 [Owens Valley Paiute]; Goodwin, 1939 [White Mountain Apache]; Jacobs, 1945 [Kalapuya]; Jacobs, 1959 [Tillamook]; Radin, 1956, p. 118 [Winnebago]; Clark, 1966, pp. 15–16 [Salishan]; Nelson, 1983, p. 18 [Koyukon]; Tenenbaum and McGary, 1984, p. 6 [Tanaina]; Sapir and Spier, 1930, p. 461 [Wishram]; Devens, 1994, p. 190 [Ojibwa]; Sobel and Bettles, 2000 [Klamath/Modoc]; Seaburg, 2007, p. 60 [Oregon Athabaskans]). These customs were probably rooted in practical motives: during daylight hours, storytelling would take time away from subsistence activities, especially during the summer. This is reflected in McClellan's observation that “wintertime was best for storytelling, since in summer everybody was busy outside” (1987, p. 248) and Reichard's observation that informants often get annoyed when linguists ask them to tell stories “in summer when there are more active things to do” (1947, p. 5; see also Steward, 1936, p. 357). Conversely, during the long winter nights, “cold and darkness restricted outdoor activities” (Burch, 2005, p. 48); consequently, people needed something with which to occupy their time, as seen in a Kalapuya informant's observation that “It is good to tell myths in the wintertime. There are long nights in the wintertime” (Jacobs, 1945, p. 51). In some cultures, these rules were reinforced with superstitions. For example, the Modoc warned that a person who told stories during the day would be bitten by a rattlesnake, and the Klamath cautioned that telling myths during warm months would cause the narrator to age more quickly (Sobel and Bettles, 2000). The Nehalem Tillamook believed that recounting myths at any time other than midwinter would bring rain or other unpleasant consequences (Jacobs, 1959, p. vii–viii), and the White Mountain Apache believed that stories should not be told during the warm months because “too much danger is abroad—snakes, poisonous insects, and lightning. For this reason people wait till such evil things are absent so they will not hear themselves spoken of and punish the narrator or his family” (Goodwin, 1939, p. vi). Even in the absence of rules, evidence suggests that nighttime is regarded as the time for storytelling: Wiessner (2014) reports that 85% of Ju/hoan nighttime conversation is dedicated to the recounting of stories and myths, as opposed to 6% of daytime conversation.

In the absence of an established system for translating the paralinguistic features of performed narrative (Tedlock, 1971; Seaburg, 2007), non-specialists are largely unaware of this important dimension of oral literature, which may explain why oral narrative has been overlooked in the natural pedagogy debate. Moreover, oral tales were often collected under atypical conditions that obscure or discourage the use of such devices. Few collectors took measures to avoid this, but there are some exceptions. For example, Scheub's collection of 10,000 audio-taped and filmed performances of Xhosa, Zulu, Swazi, and Ndebele oral narrative consists solely of performances that he did not initiate himself and “that took place normally and before audiences” (1977, p. 367). Similarly, only a handful of investigators (Reichard, 1947; Jacobs, 1959; Hymes, 1981, 2003) have attempted to document the stylistic aspects of performance, and many deliberately excluded them from their renditions, as seen in one editor's note that the translations had been “purged of the insistent repetitions and cluttering details that primitive people often stuff into their stories for ulterior purposes” (La Farge, 1963, pp. 8–9; see also Dixon, 1912, p. 2; Tenebaum and McGary, 1987, p. 3). As shown here, these “ulterior purposes” are at least in part didactic, but have heretofore been obscured by methodological missteps.

## Oral storytelling transmits generalizable knowledge

One capacity that appears to be highly developed in our species is the ability to generate and apply generalizable knowledge—that is, the “ability to generalize from familiar to unfamiliar problem-solving situations” (Greenfield and Lave, 1982, p. 192). One way in which this would have benefitted our ancestors is by enabling them to predict the location of previously unknown resource patches based on their understanding of the properties of known patches. Modern hunter-gatherers depend on this ability to locate game and other resources (Mithen, 1996). In contrast, our closest living relatives, the chimpanzees, cannot locate food patches of which they have no prior knowledge (Wrangham, 1977).

A key line of evidence that oral storytelling transmits generalizable information is the subject matter it treats. There is a marked relationship between recurrent themes in forager oral tradition and recurrent problems of forager life. For example, !Kung stories “deal with problem points in living which must always have characterized the hunting-gathering adaptation, such as uncontrollable weather, difficulty in procuring game, danger from carnivore attacks, and correct relations with in-laws” (Biesele, 1993, p. 13). Among the Ahtna, popular genres include animal myths, Ahtna history (e.g., clan origins, warfare), cultural practices (e.g., fishing, boats, copper, customs, beliefs), travel narratives, and stories about place names (Kari, 2010, pp. ix–x). Across forager oral tradition, common themes are behavioral prescriptions and proscriptions, animal habits and characteristics, warfare, famine, natural disasters, and topography (e.g., Minc, 1986; Basso, 1996; Sobel and Bettles, 2000; Barber and Barber, 2004; Ludwin et al., 2005; Scalise Sugiyama, 2011).

One of the most pervasive topics in forager oral tradition is social norms and practices. For example, Siberian Yupik tales served “to exemplify how to behave in society” (Dolitsky, 2000, p. ix). In the same vein, Alaskan Athapaskan “beliefs, attitudes, and values are passed on to new generations” through “the retelling of… stories” (Tenenbaum and McGary, 1984, p. 7). And among the Lummi, “the old people of the village relate the accomplishments of the heroes of the past in the presence of the children with the intention of educating them in tribal traditions and customs” (Stern, 1934, p. 17). Stories are also used to condemn and discourage proscribed behavior. Among the Jicarilla Apache, for example, “Should a boy be seen playing with an older sister or a female cross-cousin, a few pointed stories of mishaps likely to follow such thoughtlessness make him more discreet” (Opler, 1938, p. xii). The trickster genre is an example *par excellence* of this tactic: across forager societies, trickster is widely regarded as a model of bad behavior, and his adventures are expressly related for didactic purposes (e.g., Boas, 1898, p. 4; Thompson, 1929, p. xviii; Opler, 1938, p. 263; Street, 1972, p. 85; Biesele, 1993, p. 190; de Laguna, 1995, p. 75). For example, Yukon trickster stories “tell how Crow kept getting into trouble by wanting too much food, too much sex, and so on. Such tales kept reminding the oldtime Indians that moderation was one of their most important values” (McClellan, 1987, p. 276). Similarly, when Jicarilla elders told Coyote “tales to the children they advised them not to be foolish like this. Stories of that kind made the children think. The old people were always giving advice to the children” (Opler, 1938, p. ix).

Another widespread theme in forager oral tradition is warfare (Rink, 1875, p. 109; Parsons, 1929, p. xviii; Opler, 1938, p. v; McClellan, 1987, p. 240; Berndt and Berndt, 1994, p. 363; de Laguna, 1995, p. 289; Scalise Sugiyama, 2014). For example, Dena'ina stories include “historical accounts of events that occurred in the wars with the neighboring Yupik Eskimos.… These stories recount battles and ambushes and the deeds of heroes” (Tenenbaum and McGary, 1984, p. 7). Burch argues that, among the Iñupiaq, war narratives were an integral part of martial training: “One major element in the preparation for armed conflict was education. Stories of armed confrontations, both successful and otherwise, played a central role in Iñupiaq narrative history. By the time a person reached adulthood, he or she had heard countless stories of raids and battles as well as analyses of the strategy and tactics involved in each one” (2005, p. 72). Obviously, information about tactics, decisions, and outcomes is generalizable to other battle contexts; thus, the transmission of such knowledge could provide potentially large fitness payoffs.

Stories also transmit ecological knowledge (Scalise Sugiyama, 2011). For example, a Gwich'in informant reports that, through storytelling, “we learned about the animals that we depended on.… We also learned how to hunt the animals, what time of the year, the best locations, and the best ways to hunt” (Gwich'in Elders, 1997, p. 17). Similarly, among the Yup'ik, “vegetation, the weather, the river, local animals, etc., were all integral parts of daily life stories” (de Marrais et al., 1994, p. 205). Two genres that that recur across forager oral tradition appear to be dedicated to transmitting information about animals and topography, respectively. Etiological tales explain how animals acquired their distinctive markings or characteristics and, in so doing, describe traits that may be used to identify them from a distance and/or predict their behavior (Scalise Sugiyama, 2001). For example, a Koyukon story references the short-eared owl's habit of making repeated dives as it flies: “In the Distant Time, the short-eared owl once dropped a birchbark basket filled with fish and had to dive again and again to retrieve it” (Nelson, 1983, p. 108). Similarly, a Kalapuya story compares squirrel and deer fear responses:

Pine squirrel said to deer, “You are very much (always) afraid.… Whatever you hear, you never go look, you get up and you run to hide yourself, you run far away. You never stop (stand) in order to look back, to see what has scared you. On the other hand when I am frightened, I climb high up on a tree. Then I come back to examine, to see what has scared me. I do not always run (away). When you run, you keep running all the time, you never see what has scared you” (Jacobs, 1945, p. 132–133).

On this point, both G/wi (Silberbauer, 1981) and Shuar (Barrett, 2005) hunters are reported to use “personality profiles” (synopses of salient behavioral traits) to predict animal behavior. For example, some species are characterized as being “more skittish than others, some are more aggressive than others, and some have more sensitive smell or hearing” (Barrett, 2005, p. 215). By providing synopses of key behavioral or physical traits, etiological tales may serve to maintain and transmit these animal profiles.

Another widespread story type, the traveler-transformer tale, appears to be dedicated to transmitting topographic information. These characters are mythical, often anthropomorphized beings who traveled throughout the land long ago creating distinctive topographic features and depositing resources (Boas, 1898, p. 7). The Dreamtime is a case in point: these myths recount “the naming of places and the movements of ancestral beings from one spot to the next” (Tonkinson, 1978, p. 89). Similarly, Mohave myths were “replete with details of name and place.… In general, they described the journeys of mythical personages and told of their eventual transformation into animals or landmarks” (Stewart, 1983, p. 65; see also Kroeber, 1948, p. 3). As these observations indicate, these myths recount extensive journeys and emphasize the enumeration and description of specific sites along the way. In so doing, these stories trace travel routes, identify landmarks, and mark the location of resources (e.g., campsites, water, game, plants)—in effect providing mental maps of the audience's home range and neighboring territories. As Tonkinson explains, because many Dreamtime myths “tell of journeys covering hundreds of miles of desert, through areas that Mardudjara in many cases have not seen, they broaden the cosmological and geographical outlook of the Aborigines” (1978, p. 89). Similarly, among the Gitksan, “trails are traversed first by listening to teachings by Elders, and continue to be traveled in story as well as by actual travel on the land. The stories of the land and its named places are thus deeply enmeshed in traditional training of the young” (Johnson, 2010, p. 44). Basso (1996) describes a similar practice among the Apache. These stories can also transmit knowledge about environmental hazards and coping strategies. For example, Witsuwet'en travel narratives “mixed discussions of winter travel or travel techniques in the mountains, preventative self-discipline, and techniques for avoidance of many specific risks,” including “the need to show respect for grizzly bears or proper ax technique when clearing a winter trail of boughs and branches bent over by heavy snowfall” (Johnson, 2010, pp. 57–61).

## Discussion

The amount of teaching that occurs in forager societies may be obscured by the use of what, to Western observers, appear to be very casual modes of transmission. In industrialized societies, storytelling and conversation are viewed largely as informal or recreational activities, but in forager societies they are deliberately used for instruction. Iñupiaq teaching is a case in point:

Iñupiaq history was transmitted orally from one generation to the next in both formal and informal contexts. The formal context was the storytelling session, during which experts recounted legends and tales before assemblages made up of people of all ages.… Informal contexts included conversations and narratives delivered as people traveled about the country, crossing or passing localities of historical interest as they did so. Many historical events were recorded in place-names, of which there was an enormous number and which were known by virtually every adult. Information acquired in this way augmented and reinforced that acquired in a more formal setting (Burch, 2005, p. 48).

In Western societies, storytelling sessions are not regarded as “formal contexts,” which may feed the perception that there is “almost no direct teaching” (Blurton Jones and Konner, 1976, p. 338) and that “teaching and demonstration play a limited role” (MacDonald, 2007, p. 398) in forager societies.

Conversation is similarly underestimated as a pedagogical tool by Western researchers, despite evidence that it is an important vector of knowledge transmission among foragers (Leacock, 1954, p. 14; Laughlin, 1968, p. 308; Nelson, 1969, p. 374; Biesele, 1978, p. 940). For example, among the Ache, “each man usually reports to the others in considerable detail concerning every game item that he encountered that day, and the outcome of the encounter” (Kaplan and Hill, 1992, p. 196). And in a series of animal behavior “seminars” conducted with !Kung hunters, Blurton Jones and Konner found that “the participants seemed to gain a lot of new information, or at least heard about observations and generalizations concerning behavior which were quite new to them” (1976, p. 338). They concluded that the acquisition of hunting knowledge “appears to be quite simply a matter of watching and *listening to other people* and then trying for one's self” (1976, p. 338; emphasis added).

On this point, indigenous informants commonly report that stories are used for educational purposes. For example, Gwich'in Elder Mary Kendi notes that, “by telling the stories, the Elders and our parents were able to pass on their knowledge and the knowledge of our ancestors” (Gwich'in Elders, 1997, p. 17). Similarly, a Jicarilla Apache man reports that “They told the children to pay attention, that these stories would show them what to believe and learn” (Opler, 1938, p. ix). Moreover, storytelling is explicitly characterized as a form of teaching, as seen in Mary Kendi's observation that, “as I grew older and had my own family I had to teach my children about the land and animals. I became the story teller, the teacher” (Gwich'in Elders, 1997, p. 17). This claim is echoed by ethnographers. For example, in her history of Yukon peoples, McClellan notes that the “elders who contributed to this book also often remarked on how people trained themselves in the old days to remember what they heard, because it was by listening to stories and retelling them that the Indians learned and passed on much of what they knew about the world” (1987, p. 252; see also Clark, 1966, p. xv). Similarly, among the Ju/'hoansi, “many sorts of knowledge are acquired (by young and old Ju/'hoansi alike) through hearing the dramatized story of a day's events rather than in a directly didactic learning context” (Biesele, 1993, p. 43). In the same vein, Ojibwa “storytelling was an important part of teaching children.… about nature or proper behavior” (Devens, 1994, p. 190).

Because studies of forager pedagogy do not typically take storytelling into account, we might have a false picture of the amount of verbal instruction that occurs among foraging peoples. For example, in an observational study of teaching interactions between Aka caregivers and 12–14-month-old infants, Hewlett and Roulette (2016) found that verbal instruction was rare. However, it is possible that, in forager societies, verbal instruction occurs largely in the form of storytelling and that storytelling is directed at older children. If this is the case, we would not expect to find much verbal instruction directed at infants and, conversely, we would expect to find practices aimed at facilitating and/or testing narrative fluency in older children. Tellingly, there is evidence that, in some forager groups, children were taught stories though rote memorization. For example, the Nehalem Tillamook held special storytelling sessions for children in which tales were “recounted for the purpose of teaching children to tell the stories verbatim” and “the child repeated each sentence after the raconteur” (Jacobs and Jacobs, 1959, p. vii–viii). Similarly, among the Dena, tales were re-told by the same narrator in order to “fix it in the memory of the listeners,” whose ability “to repeat the story accurately, and honestly (without help or hints), would be tested later by their elders” (de Laguna, 1995, p. 74–75).

Interestingly, Hewlett and Roulette's operationalization of verbal instruction as the provision of “some verbal explanation… about a task or knowledge” (2016, p. 6) is an apt definition of the manner in which “how-to” information is presented in oral tradition. For example, in a Yokuts story, Jack Rabbit tells the people how to make fire if they lack buckeye wood for making fire drills: he “told them about a certain kind of white rock; he said to save it whenever they found it. By striking these rocks together over pieces of inner bark from the oak, they could always make fire” (Gayton and Newman, 1940, p. 21). Unlike written communication, oral storytelling lends itself to demonstration, which is sometimes detectable in transcribed texts. For example, a Yamana story that describes a character making fire notes that he “stood in such a way,” suggesting that the narrator was indicating with his body how to position oneself for this purpose:

When he struck this stone against another a small spark came out.… Hurriedly, he took a handful of very dry down. These he put on the ground. Once more he began to draw a spark from the š*ewáli*. He stood in such a way, striking in a certain direction, that the spark sprang into the down. The down began to glow, caught fire, and soon a small flame leaped up. Yoálox quickly went to fetch kindling and wood and put it on the flame (Gusinde, 1977, p. 31).

Practical information of this sort is often incidental to the plot, or may appear so from an etic perspective. As Biesele notes, “Much subsidiary information about the behavior of animals, the habitats of plants, and the customs of humans regarding them is tucked within the artistry of stories” (1993, p. 45). Origin stories are a case in point, presenting information about “how to” in the course of explaining “wherefore.” As Ramsey explains, “To know how Coyote first caught and prepared salmon is to know how these crucial acts are to be performed” (1977, p. xxiii). Similarly, information about the best materials for making fire is often couched in myths about an elaborate ruse used to steal fire from beings who are monopolizing it (e.g., Lowie, 1954; Kroeber and Gifford, 1980). As a result, generalizable information can easily escape the notice of Western investigators, which may further bias estimates of the amount of verbal teaching that occurs in forager societies.

Teaching may also be masked by the disinclination to present educational content overtly. For example, “As a rule, Aborigines seemingly preferred their mythology not to be *directly* censorious” (Berndt and Berndt, 1994, p. 3). Similarly, Ju/'hoansi tales “are ‘didactic,’ but only by indirection; they outline the consequences of social action rather than preach” (Biesele, 1993, p. 182). Likewise, “the didactic content of Zuñi tales is usually either implicit or addressed by one tale character to another, and is never addressed by the narrator directly to his audience” (Tedlock, 1971, p. 117). As Tedlock explains, the narrator's interpretation of the story is woven into the delivery: “When I do my version of a story… I give my *interpretation*. I do not say, ‘I think that the protagonist is to blame for what is happening to him,’ but I may *portray* the protagonist in that way. If someone likes my version… he will emulate it; if not, he will tell the story in his own chosen way” (1971, p. 516, emphasis in original). This may be related to the general aversion to direct instruction that has been documented in a number of forager societies (e.g., Gould, 1969; Lee, 1969), and is expressed as disapproval of “people who tell other people what to do” (Blurton Jones and Konner, 1976, p. 345).

Finally, another factor that may obscure the amount of teaching that occurs in forager societies is information ownership. In Arnhem Land, for example, “myths were… a form of property; as such, they could be inherited or stolen, or legitimately used only with the owner's permission” (Allen, 1975, p. 23; see also Radin, 1956, p. 122; Ludwin, 2002). Greenland Inuit traditions of knowledge ownership illustrate how this practice may constrain transmission:

Ownership applied… to certain kinds of information that were revealed only for a fee.… Specialized training was provided by different persons, each recognized as an authority on a particular topic. One man listed seven major categories of specialized knowledge: hunting, kayaking, feats of strength, drum singing, magic prayers, witchcraft, and shamanistic practices. Each included subdivisions; for example, hunting instructions were for specific species and were offered by the persons most adept at taking them. It appears that the teacher was always an older person, and he usually offered individual instruction. A tyro might have numerous instructors about the same subject, especially for dealing with the supernatural (Oswalt, 1999, p. 154).

If instruction took place largely on an individual basis, it would be difficult to detect ethnographically. Moreover, some types of instruction, such as hunting and kayaking lessons, necessarily take place away from camp, which would further reduce the chances of their being observed by ethnographers.

In sum, when testing for the presence of pedagogy in forager societies, it may be best to discard Western notions of “direct teaching” and look instead for evidence of ostensive communication. Birket-Smith's (1929) discussion on this point is illuminating:

One cannot say that there is a lack of *purposeful* education among the Caribou Eskimos, as the parents little by little teach their children what they think is right. It might rather be said that there is a want of *systematic* education, but I am not sure even of that, for the necessary manual accomplishments and moral principles… are gradually impressed upon the children (1929, p. 288, emphasis in original).

As Csibra and Gergely argue, the “function of pedagogy is to allow transfer of culturally accumulated knowledge to new members of the community” (2006, p. 11). Oral storytelling appears to meet these criteria: its abundant use of ostension attests to its pedagogical intent, and predictable, cross-cultural patterns in certain types of content attest to its transmission of generic knowledge. Coupled with the fact that storytelling is a human universal (Brown, 1991), the research reviewed here supports the hypotheses that teaching is a reliably-developing feature of our species and that, in our ecological niche, much learning is “a matter of listening to people story-telling, and not a more highly ordered system of information transmission” (Blurton Jones and Konner, 1976, p. 342).

## Author contributions

The author confirms being the sole contributor of this work and approved it for publication.

### Conflict of interest statement

The author declares that the research was conducted in the absence of any commercial or financial relationships that could be construed as a potential conflict of interest.
